# Radial head stabilization after ulnar lengthening in type IIB multiple hereditary exostoses: a pediatric case report using a limb reconstruction system and fixation button

**DOI:** 10.1097/RC9.0000000000000437

**Published:** 2026-04-08

**Authors:** Patar Parmonangan Oppusunggu, Gabriel Klemens Wienanda, Mitchel Mitchel, Karina Sylvana Gani, Erica Kholinne

**Affiliations:** a Department of Orthopedics and Traumatology, Gatam Institute, Eka Hospital, Tangerang, Indonesia; b Department of Orthopedics and Traumatology, Eka Hospital Cibubur, Bogor, Indonesia; c Department of Surgery, Faculty of Medicine, Universitas Trisakti, Jakarta, Indonesia

**Keywords:** case report, fixation button, gradual ulnar lengthening, LRS, multiple hereditary exostoses, radial head dislocation

## Abstract

**Introduction:**

Multiple Hereditary Exostoses (MHE) is a genetic disorder causing multiple osteochondromas, often affecting the forearm and leading to ulnar shortening and radial head dislocation. Surgical management in children is challenging, especially in achieving stable reduction.

**Case presentation:**

An 8-year-old girl with a 3-year history of left elbow deformity and restricted motion who underwent gradual ulnar lengthening at a rate of 0.25 mm/6 hours, achieving a total distraction of 3 cm with overlengthening by 1 cm, followed by stabilization using a fixation button technique to allow spontaneous radial head reduction, ensuring stable alignment without the need for open reduction. At three months, the patient achieved full, pain-free motion of the elbow and forearm, with no re-dislocation observed. Importantly, no perioperative complications, including pin site infection or neurovascular compromise, were encountered throughout the healing process.

**Clinical discussion:**

Combined ulnar lengthening and fixation button stabilization effectively maintained radial head reduction and improved early functional outcomes.

**Conclusion:**

This case highlights a novel application of fixation button fixation following gradual ulnar lengthening to prevent radial head re-dislocation in a pediatric patient with MHE Type IIB. This approach successfully restored joint alignment and function, offering a potential surgical option in similar complex cases.

## Introduction

Multiple Hereditary Exostoses (MHE) is an autosomal dominant condition marked by the presence of multiple osteochondromas on bones that develop through endochondral ossification^[^1^]^. These lesions form due to abnormal chondrocyte activity at the growth plate, leading to herniation of cartilage through the periosteum^[^2,3^]^. As a result, various deformities may occur, including ulnar shortening, bowing of the forearm bones, increased ulnar tilt of the distal radius, ulnar deviation of the hand, progressive carpal translocation, and radial head dislocation^[^4^]^. These structural changes can cause wrist instability, pain, reduced grip strength, and limited forearm rotation^[^5^]^. Radial head re-dislocation is more common in younger patients, with an incidence of approximately 8.3%^[^6^]^.

HIGHLIGHTS

- Radial head dislocation in MHE commonly results from progressive ulnar shortening and compensatory radial bowing.
- Early detection and timely intervention are essential to prevent irreversible deformity and functional loss.
- Using a fixation button offers a less invasive way to maintain radial head stability while preserving rotational motion.
- Performing surgery earlier in growing children may help prevent worsening deformity and improve overall function.

There is currently no consensus on the optimal surgical strategy for MHE-related radial head dislocation in children. Ulnar lengthening alone may provide favorable outcomes when dislocation or severe deformity is absent, but established dislocation makes stable reduction and correction of secondary deformities more challenging^[^7^]^. Surgical options include ulnar osteotomy with or without gradual distraction, the use of an Ilizarov circular external fixator for multiplanar correction, or monolateral fixators such as the Limb Reconstruction System (LRS), which offer simpler construction and improved patient comfort. The choice of technique depends on patient age, severity of deformity, and surgeon preference^[^8^]^.

Based on our knowledge, there is limited literature regarding the use of fixation button following gradual ulnar lengthening to prevent radial head redislocation in pediatric patients with MHE. We report the case of a pediatric patient with radial head dislocation secondary to MHE who was treated with gradual ulnar lengthening combined with fixation button. The current study has been reported in accordance with SCARE 2025 standards^[^9^]^.

## Case presentation

**Figure 1.**
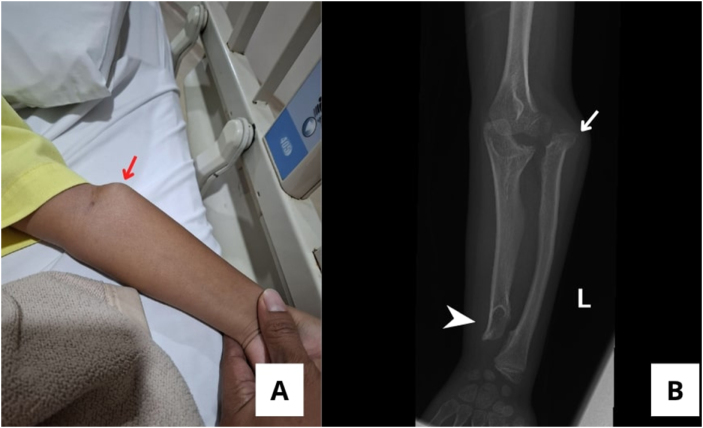
(A) A pre-operative clinical presentation showing a small, firm, and non-tender nodule in the left elbow (red arrow). (B) Plain radiograph AP view of the left forearm showing anterolateral dislocation of the radial head (white arrow) and a shortening of the ulnar (white head arrow).

An 8-year-old girl presented to the orthopedic clinic with a 3-year history of left elbow deformity without pain or sensory symptoms. She had no history of trauma or infection and was previously diagnosed with a benign bone neoplasm. Examination showed restricted elbow motion with flexion–extension limited to 10°, and inability to pronate or supinate. A firm, non-tender nodule was palpable at the elbow. Pre-operative radiographs revealed ulnar shortening with distal bumps of the radius and ulna, and anterolateral displacement of the radial head, consistent with neglected radial head dislocation secondary to MHE (Fig. 1).

**Figure 2.**
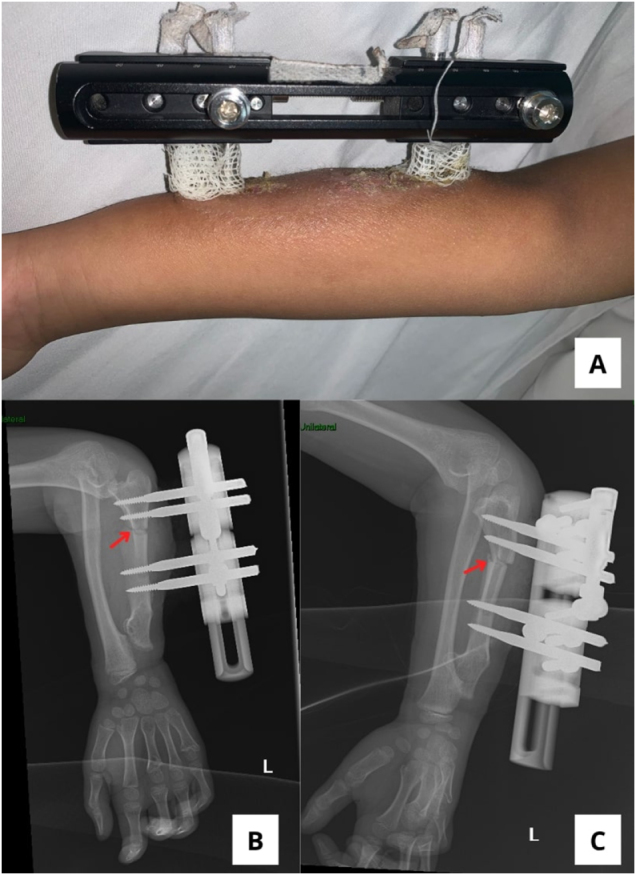
Immediately post-operative shows (A) LRS device was successfully applied. Plain radiograph of forearm (A) AP view and (C) lateral view shows signs of osteotomy (red arrow) and LRS devices on the left arm.

**Figure 3.**
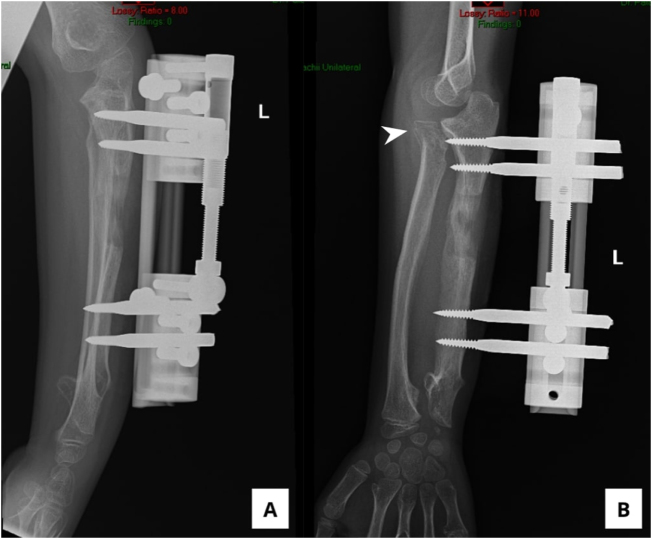
Two months post-operative plain radiograph of left forearm (A) AP view shows callus bridging; (B) lateral view shows radial head reduction (white head arrow).

The first surgery was performed in February 2025. The details of the timeline of this case can be seen in Table 1. The plan included radial head reduction and gradual ulnar lengthening using a pediatric Limb Reconstruction System (LRS) implant (PT ENDO – LRS Pediatric). Ulnar osteotomy was performed, and the LRS was applied (Fig. 2A–C). Distraction was initiated at 0.25 mm every 6 hours for 4 weeks, followed by 4 weeks until radiological signs of new bone regeneration and callus formation were observed. The patient was monitored weekly for fixator stability, pin-site care, and neurovascular status. She remained pain-free, with elbow flexion up to 95° and the presence of wrist pronation and supination observed after the first surgery during the distraction period. At 2 months, clinical assessment showed full wrist motion, further improvement of pronation–supination, and improved elbow ROM (0–95°) without pain. Radiographs confirmed 3 cm of new bone formation and maintained radial head reduction (Fig. 3A and B).

**Table 1:** Chronological summary of patient evaluation and management.

Date	Description/procedures	Physical findings	Imaging
January 28th, 2025	The patient comes to the orthopedic outpatient clinic	Unable to pronate or supinate her forearm Flexion and extension of the elbow were restricted to 10 degrees. A small, firm, non-tender nodule was palpable over the left elbow (Fig. 1A)	Fig. 1B
February 3rd, 2025	1st operativeUlnar osteotomy and implanting LRS in the left forearm.	Same as January 28th, 2025	Fig. 2A
February to April 2025	Gradual lengthening of the ulna to 3 cm	The patient did not report any pain or sensory disturbances	Fig. 2B and 2C
April 5th, 2025	Gradual lengthening completed	Normal range of pronation and supination Flexion and extension of the elbow were improved to 0 to 95 degrees No palpable nodule	New bone formation is observed, and the radial head remains fully reduced in its anatomical position. (Fig. 3A and B)
	2nd operative LRS was removed, followed by the placement of a fixation button between the radius and ulna, and the application of a cast for immobilization.		Fig. 4
April 19th, 2025	The cast was removed	Normal range of pronation and supination Normal range of flexion and extension	None
July 22nd, 2025	3 months post-operatively	Normal range of pronation and supination Normal range of flexion and extension No complaint and no radial head redislocation observed	None

**Figure 4.**
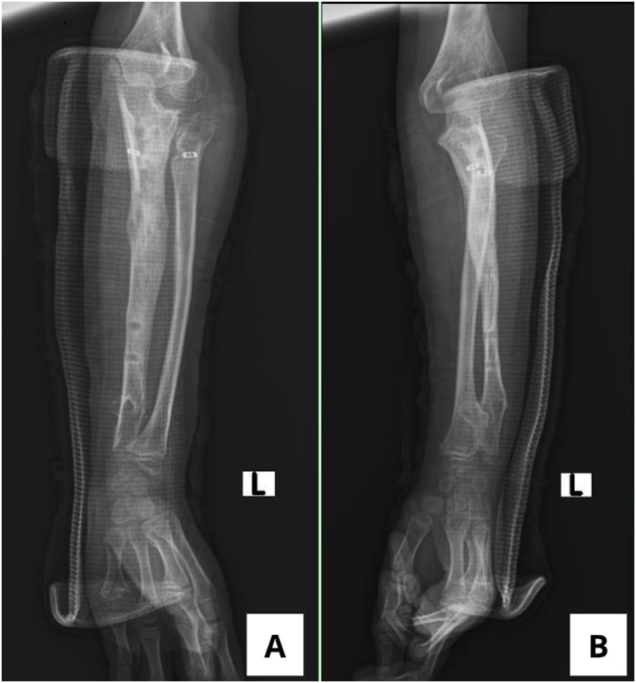
Post-secondary operative X-ray of the left forearm. (A) AP view and (B) lateral view show bone formation of the ulnar, and the fixation button was confirmed to be in the correct position.

In May 2025, the second surgery was performed, involving the removal of the LRS and the insertion of a Knitrope fixation button (Doratek Medikal, Istanbul, Turkey) from the proximal radius to the proximal ulna, placed 0.5 cm below the annular ligament. This was intended to reduce the risk of radial head redislocation while preserving motion and function. The elbow was immobilized in a cast for 2 weeks. Postoperative radiographs showed correct button placement and no redislocation (Fig. 4A and B).

At 2 weeks, the cast was removed, and the patient achieved full elbow, wrist, and forearm ROM. At the three-month follow-up, she remained asymptomatic, with stable reduction and no complications, infections, or neurovascular deficits.

## Discussion

This case demonstrates the combined use of gradual ulnar lengthening with a Limb Reconstruction System (LRS) and fixation button stabilization to manage a type IIB forearm deformity in a pediatric patient with multiple hereditary exostoses (MHE). Type IIB deformities are complex and often limit the effectiveness of standard stabilization techniques. By integrating soft-tissue stabilization with bony correction, this approach aimed to enhance joint stability and minimize redislocation. Our findings underscore the potential advantages of combining soft tissue stabilization with bony correction in managing complex MHE cases.

MHE is an autosomal dominant genetic disorder characterized by multiple osteochondromas, with a prevalence of about 1 in 50 000 live births and forearm involvement in 30–74% of cases^[^1,4^]^. Approximately 80% of patients become symptomatic within the first decade of life. Although earlier surgery, before radial head dislocation has been associated with better aesthetic outcomes^[^6^]^. Our patient achieved both excellent cosmetic and functional results despite undergoing surgery after dislocation had already occurred.

**Figure 5.**
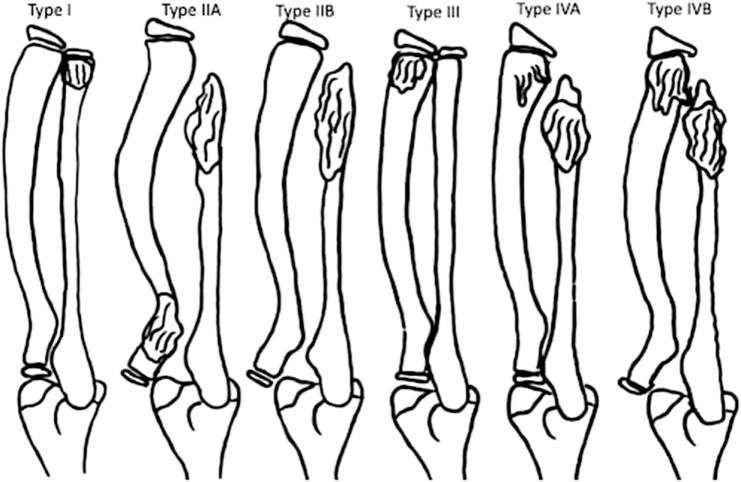
Classification of MHE of the forearms^[^10^]^. Type I: distal ulnar exostosis, Type IIA: distal ulnar and proximal radial exostosis with radial head dislocation; Type IIB: distal ulnar exostosis with radial head dislocation; Type III: distal radial exostosis; Type IV: distal radial and ulnar exostosis; Type IVA: without radial head dislocation; Type IVB: with radial head dislocation.

In this study, the MHE cases could not be classified using the standard Masada system, so a modified Masada-based classification was applied. According to Jo et al. classification relies on exostosis location, which influences deformity severity^[^10^]^. Our patient was categorized as type IIB, characterized by distal radial and ulnar exostoses with radial head dislocation (Fig. 5). The distal ulnar growth plate is smaller than that of the radius, making it more susceptible to growth disturbance from an exostosis. This disruption causes relative ulnar shortening, prompting compensatory radial bowing that can progress to radial head subluxation or dislocation^[^11^]^.

We used a pediatric monorail external fixator (LRS), placed laterally and parallel to the ulna, with four Schanz screws to ensure lengthening along the ulna’s anatomical axis. The device did not cross the wrist or elbow, allowing full motion during lengthening. However, Song et al. reported that although the modified Ilizarov technique can correct MHE-related forearm deformities, recurrent ulnar shortening may still occur in growing children^[^4^]^. Gradual ulnar lengthening can place tension on nearby neurovascular structures, particularly the ulnar nerve and surrounding vessels. In our case, weekly evaluations of pin sites and neurovascular status were performed. Distraction osteogenesis protocols emphasize frequent neurovascular monitoring to prevent complications^[^6,8^]^, and large pediatric series note pin-site infection as a common issue requiring vigilance^[^12^]^. Refsland *et al* also reported no neurovascular complications in MHE correction^[^6,7^]^.

Ulnar overlengthening of 0.5–1.0 cm in skeletally immature patients can reduce recurrence and is generally well tolerated due to children’s high remodeling capacity and continued radial growth^[^7^]^. Pediatric patients are also less prone to degenerative changes from temporary overlengthening^[^9^]^. However, Matsubara noted that outcomes are unpredictable and excessive lengthening may cause ulnocarpal impaction^[^13^]^. In this case, the ulna was lengthened 3 cm, including 1 cm of overlengthening, with good recovery, and a fixation button was added to prevent recurrence. Although some authors recommend delaying surgery until after age 10 or adolescence^[^14^]^, early intervention may better prevent progression and support radial head reduction^[^15^]^.

In this patient, a fixation button was applied after ulnar lengthening to maintain joint stability and limit radial head overcorrection. Wang et al. similarly used anchors with absorbable sutures to reinforce stability and prevent redislocation^[^16^]^. When radial head dislocation persists despite corrective procedures and leads to pain or functional decline, salvage or reconstructive options may be considered^[^17^]^. Although ulnar callotasis can improve alignment, it may not correct longstanding radial head dislocation, as shown by Vogt et al. in pediatric MHE patients^[^7^]^. Likewise, Kumara et al. reported persistent dislocation in some Masada IIB cases despite reconstruction^[^18^]^. Fixation button stabilization offers a minimally invasive, motion-preserving method following successful reduction, whereas annular ligament or tendon-graft reconstructions are typically reserved for recurrent instability and carry higher operative demands^[^19^]^. External fixation poses risks such as pin-site infection, influenced by pin characteristics and care quality, though antibiotic injections can reduce infection rates^[^20^]^. In our case, no pin-site infection, neurovascular issues, premature consolidation, or radial head redislocation occurred during the three-month follow-up.

The fixation button technique offers several advantages compared with annular ligament reconstruction or tendon graft stabilization, including minimal soft-tissue disruption, preservation of forearm rotation, shorter operative time, and reduced surgical morbidity, particularly in skeletally immature patients^[^16^]^. Key technical pearls include precise anatomical localization, careful drill orientation, and maintenance of forearm supination throughout the procedure. However, the technique is technically demanding and carries several pitfalls, such as posterior interosseous nerve (PIN) injury due to improper tunnel placement, excessively distal positioning of the button, uncontrolled drill trajectory, or aggressive soft-tissue dissection, all of which may increase neurovascular risk^[^21,22^]^. In the present case, several technical precautions were applied to minimize these risks. The fixation button was positioned approximately 0.5 cm distal to the annular ligament through limited and meticulous soft-tissue dissection, avoiding circumferential exposure of the radial neck. The forearm was maintained in supination during drilling, a position known to displace the PIN away from the operative field. Blunt dissection to bone, controlled drill trajectory close to the cortex, and avoidance of deep or blind instrument passage were emphasized. No aggressive retraction was used, and postoperative clinical evaluations demonstrated no motor or sensory deficits, indicating effective neurovascular protection. Despite its advantages, potential disadvantages include technical sensitivity, dependence on surgeon experience, and uncertainty regarding long-term stability during continued skeletal growth, which warrant cautious application and further study.

The main limitation of this study is the short follow-up of only three months. The patient’s residence, located in a remote area with limited access to specialized care, imposed restrictions on continued monitoring due to logistical and financial constraints. Although long-term follow-up is ideal, these barriers prevented regular assessment. Detailed discharge instructions and recommendations for local evaluations were provided, with plans for reassessment if the patient becomes more accessible in the future.

## Conclusion

This case demonstrates successful management of a type IIB forearm deformity in pediatric MHE using staged ulnar lengthening and fixation button stabilization. While effective, recurrence remains a concern in skeletally immature patients, underscoring the need for early intervention and vigilant long-term follow-up until skeletal maturity.

## Data Availability

No datasets were generated or analyzed for this study.
